# Drug Release Property of Poly 3-Hydroxybutyrate 4-Hydroxybutyrate (P34HB) as Drug-Eluting Coatings on Metal Coronary Stents

**DOI:** 10.3390/polym14153018

**Published:** 2022-07-26

**Authors:** Yihui Jian, Yufang Zhu

**Affiliations:** 1 School of Materials and Chemistry, University of Shanghai for Science and Technology, Shanghai 200093, China; 2 AccuPath Medical (Jiaxing) Co., Ltd., Jiaxing 314006, China; 3 Shanghai Institute of Ceramics, Chinese Academy of Sciences, Shanghai 200050, China; zjf2412@163.com

**Keywords:** poly 3-hydroxybutyrate 4-hydroxybutyrate (P34HB), drug coatings, drug release, coronary stents, DES

## Abstract

Drug-eluting stents (DES) have become the main method of interventional therapy for coronary heart disease, because their drug coating can effectively reduce the incidence of restenosis after stent implantation. Biodegradable polymers for coatings are the latest development direction for coating polymers, because they can be degraded into small molecules in the human body. In this study, the polymer P34HB(P34HB-1:4HB% = 1 mol%, Mw: 225,000; P34HB-10:4HB% = 10 mol%, Mw: 182,000), the fourth generation of biodegradable Polyhydroxy alkanoates (PHAs), was coated on stents to evaluate the drug release properties of the DES. Both P34HB-1 and P34HB-10 coatings showed increased drug release rates, as the polymer concentrations were gradually increased from 8 mg/mL to 28 mg/mL. Both P34HB-1 and P34HB-10 coatings showed increased drug release rates as the drug polymer ratios were gradually changed from 1:10 to 1:2. The drug release rates of the P34HB-1 coatings became slower than P34HB-10, thus showing sustained drug release effects. The drug release rates of the P34HB-1 coatings decreased when Rates of solution flow increased, decreased when Focusing pressures decreased, and decreased when Mandrel moving speeds increased. P34HB-1 coatings prepared with CHCl_3_/NPA (10:1) mixed solvents had better controlled drug release rates compared to Firebird2^®^. The drug release rates of P34HB-1 coatings prepared with CHCl_3_ solutions decreased as the outer layer weights were increased from 0 to 800 μg. When the outer layer weights reached 800 μg, the drug release rates of P34HB-1 coatings were slower than Firebird2^®^. P34HB-1 coatings prepared with both CHCl_3_/NPA (10:1) mixed solvents and double layers had more effectively controlled drug release rates than P34HB-1 coatings prepared with only mixed solvents or double layers and these effects were far greater than Firebird2^@^; thus, P34HB-1 represents a latent polymer for DES.

## 1. Introduction

*The 2019 Global Health Estimation* released by the World Health Organization points out that coronary heart disease has been the leading cause of death in the world over the past 20 years and now accounts for 16% of all deaths. It is common in older persons and has also tended to occur in younger people in recent years [1].

DES interventional therapy is the most important treatment for coronary artery stenosis. There are two main kinds of coating polymers for coronary stents: non degradable, and degradable. Biodegradable polymers are hydrolyzed or enzymatically hydrolyzed and degraded into small molecular substances such as CO_2_ and H_2_O, after being implanted into blood vessels. These substances are discharged through urinary and other excretory systems from the body, without cumulative toxicity, so the safety is improved [2].

Degradable coating polymers are the mainstream of DES. At present, some drug stents in the market use PLA polymer, but this polymer tends to rupture and can be peeled from the bare stents after the balloon expansion, because of its low ductility [3] and insufficient flexibility [4].

Natural PHAs are biodegradable and they have been studied extensively for various potential medical applications [5,6]. PHAs include the first generation PHB, the second generation PHBV, the third generation PHBHHx, and the fourth generation P34HB. At present, most of the researches on the biomedical field of drug release are concentrated on the first and second generations, with some on the third generation, while research on the fourth generation, especially the research on drug stents, is almost absent. For example, Bazzo et al. [7] prepared PHBV/PLA microspheres containing ibuprofen, to prolong the drug release and in vitro dissolution profiles, and showed that the formulation containing PHBV/PLA at the proportion of 30/70 gave the best results, in terms of prolonging the ibuprofen release. Li et al. [8] prepared composite microspheres from bioactive wollastonite (W) and PHBV. The results showed that in the phosphate buffered saline (PBS) and modified simulated body fluid (SBF) solutions, gentamicin was released from the PHBV/W composite microspheres at a relatively lower rate compared to that of the pure PHBV microspheres, and 90% of the total amount of gentamicin was released from the composite microspheres after soaking for 22 days, which was much longer than that for the release of the same amount gentamicin from the pure PHBV microspheres (8 days). This showed that the PHBV/W composite microspheres might be applied as the controlled drug release systems. Wang et al. [9] prepared PHBV/Hydroxyapatite (HA) composite microspheres as a long-term drug delivery system, and its sustained release lasted more than 10 weeks. The system showed a very low initial burst, which could be neglected, owing to the high affinity and absorbability of nano-HA particles. The drug release rate in vitro was controlled by the diffusion rate of the drugs from the polymer matrices. Thus, PHBV/HA composite microspheres could be a promising long-term drug release system. Peng et al. [10] designed implantable sandwich PHBHHx films, to prolong the release time and to inhibit the burst release phenomenon of thymopentin (TP5), using a simple volatilization method. In vitro release studies revealed that the sandwich films had nearly no burst release. The in vivo release time of the sandwich films was prolonged to 42 days. Thus, sandwich PHBHHx films show excellent potential as a sustained, burst-free release system for small-molecular-weight, hydrophilic peptide drugs.

Since PHBHHx is much softer than P34HB (fourth-generation of PHAs) because of its side-chains of C_3_H_7_ which was not found in P34HB, drugs may be much easier to diffuse through the polymer coatings into the media. It can be inferred that P34HB biodegradable polymers may have better controlled drug release rates than PHBHHx and may be used as latent coating materials. To date, there has been no research reports on P34HB as a drug release coating material for coronary stents. Therefore, it is necessary to further study the drug release performance of P34HB.

Previously, relevant research on P34HB for coronary drug-eluting stents has been completed [11]: Using P34HB-1 (4HB% = 1 wt%, Mw: 225,000) and P34HB-10 (4HB% = 10 wt%, Mw: 182,000) as two candidates, both P34HB-1 and P34HB-10 exhibited excellent solubility in CHCl_3_. Their drug solutions remained highly stable and did not become turbid over a period of 48 h and were conducive to batch preparation of uniform drug coatings. Drug coatings made with both P34HB-1 and P34HB-10 on stents were almost complete before and after dilation by balloon, owing to their excellent adhesion and extrusion resistance properties. Both P34HB-1 and P34HB-10 had excellent biocompatibility in cytotoxicity and hemolysis tests. P34HB-1 drug coatings showed better drug release control than P34HB-10 drug coatings and Firebird2^®^ while using mixed solvents.

In this study, fourth-generation P34HB polymers(P34HB-1:HB% = 1 wt%, Mw: 225,000 and P34HB-10:4HB% = 10 wt%, Mw: 182,000) were utilized as coating polymers for drug-eluting stents. The effects of P34HB solution formulations, spraying parameters, mixed solvents, and coating structures on the drug release rates of the coatings were systematically studied in this paper.

## 2. Materials and Methods

### 2.1. Materials

Bare stainless-steel stents were prepared by laser cutting of small tubes, followed by electrochemical polishing. P34HB polymers(P34HB-1:4HB% = 1 wt%, Mw: 225,000; P34HB-10:4HB% = 10 wt%, Mw: 182,000) were purchased from Bluepha Co., Ltd. (Beijing, China). Sirolimus (RAPA, HPLC grade) with a purity greater than 98wt% was purchased from Shanghai Jiahe Biological Technology Co., Ltd. (Shanghai, China). Both CHCl_3_ and N-propanol (NPA) were HPLC grade and purchased from Sinopharm Group (Shanghai, China).

### 2.2. Coating Procedure

Polymer coatings on the stents were prepared using the ultrasonic spray-coating method of Sono-tek Corporation. The P34HB solutions containing RAPA were prepared and injected into the syringe pump before being spraying. The drug stents were taken out and dried at room temperature for at least 24 h after the ultrasonic spray coating procedure [12].

### 2.3. Drug Release Profiles Measurement

First, 15 mL PBS/ethanol (9/1, pH = 7.4) solution was placed into a thermostatic oscillator at 37 °C and 80 rpm to reach a constant temperature, for evaluation of in vitro drug release; then, one piece of DES was immersed in the solution for 28 days. The solution was replaced with a fresh solution at 3 h, 24 h, 7 days, and 14 days. A HPLC machine (Agilent series 1100, Santa Clara, CA, USA) was used to test the drug content of each replaced solution. Accumulated release rates at the above times were calculated using (1). The initial drug content of a drug-eluting stent was calculated using the coating weight and drug polymer ratio. Three pieces of DES as a group were made for the drug release profile measurements.

Accumulated release rate (%) at time x = (total drug contents of all replaced solutions at time x/initial drug content of a drug-eluting stent) × 100%(1)

### 2.4. DOE

The DOE of the three main influencing factors (Rate of solution flow; Mandrel move speed; Focusing pressure) for the response variable (24 h drug release rate) was introduced. Statistical tools were used to analyze the data, find an effective fitting mathematical model, and obtain the optimal spraying parameters and the optimal drug release value.

### 2.5. Polymer Crystallinity Measurement

#### 2.5.1. Samples Made for Testing

All groups (a: 1.8 g P34HB-1, 100 mL CHCl_3_; b: 1.8 g P34HB-1, 100 mL CHCl_3_/NPA (100:5); c: 1.8 g P34HB-1, 100 mL CHCl_3_/NPA (10:1)) were stirred for several minutes until clear. The P34HB-1 films stayed at the bottom of the beakers after the solvents had evaporated. Finally, each piece of P34HB-1 film was cut into three pieces with a thickness of about 1 mm.

#### 2.5.2. Crystallinity Measurement

Crystallinities of P34HB were measured by an X-ray diffractometer (Rigaku Ultima IV).

### 2.6. Polymer Viscosity Measurement

#### 2.6.1. Samples Made for Testing

All groups (a: 1.8 g P34HB-1, 0.18 g RAPA, 100 mL CHCl_3_; b: 1.8 g P34HB-1, 0.18 g RAPA, 100 mL CHCl_3_/NPA (100:5); c: 1.8 g P34HB-1, 0.18 g RAPA, 100 mL CHCl_3_/NPA (10:1)) were stirred for several minutes until clear. Each sample was divided into three parts for testing.

#### 2.6.2. Viscosity Measurement

Viscosities of P34HB-1 solutions were measured by a BROOKFIELD Digital Viscometer (LVDV-C type).

## 3. Results and Discussion

When a stent is implanted into a blood vessel, the drug loaded in the polymer coating of the metal stent is eluted and released around the blood vessel wall tissue, to form a high concentration of drug, which can effectively prevent the restenosis caused by the scratching of the blood vessel wall after implantation. In this study, the effects of P34HB solution formulation, spraying parameters, mixed solvents, and coating structure on coating drug release rates were analyzed, as in the following:

### 3.1. Effects of Polymer Concentrations Prepared for Coatings on Drug Release Rates

**Figure 1:**
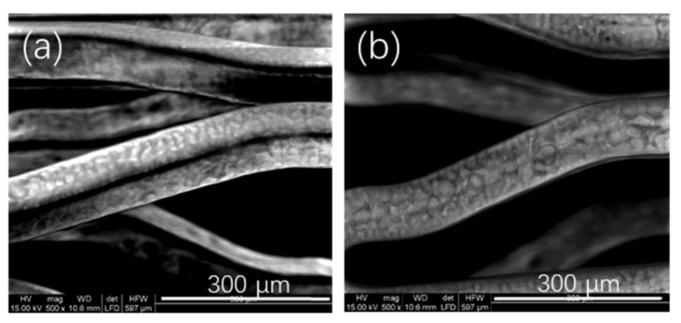
Morphologies of coating surfaces made by different concentrations of P34HB. (**a**) 28 mg/mL; (**b**) 30 mg/mL.

The experiment in this section was to investigate the effects of spraying solutions with different P34HB concentrations prepared for coatings on drug release rates, while these solutions retained the same drug polymer ratios (such as RAPA:P34HB = 1:10). When the P34HB concentrations reached 30 mg/mL (Figure 1b) or even more, the coatings on the stent surfaces began to become rough during spraying. When the P34HB concentrations were 28 mg/mL (Figure 1a) or less, the coatings on the stent surfaces began to become smooth. Therefore, the solution concentration range selected here was within 28 mg/mL.

**Figure 2:**
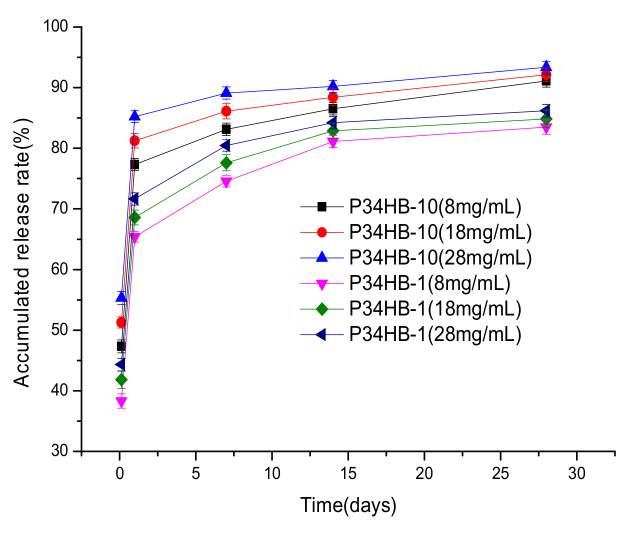
Drug release profiles of coatings with different P34HB concentrations.

Figure 2 shows that both the P34HB-1 and P34HB-10 coatings’ drug release rates gradually became faster and faster with time; at first, evidently increasing, especially within 24 h, then increasing smoothly till 28 days. Previous experiments [11] explained that the main reason for this may be that the diffusion control and polymer swelling took effects according to P34HB’s fitted drug release model At the initial stage, the drug concentrations were high, and the drug release rates were fast after polymer swelling. At the later stage, the drug concentrations were low, and the drug release rates were slow.

Both the P34HB-1 and P34HB-10 coatings showed increased drug release rates as their polymer concentrations were gradually increased from 8 mg/mL to 28 mg/mL. The main reason for this may be that, as the concentrations of polymer solution increased, the degree of entanglement between the polymer chains increased. The greater the degree of such entanglements, the less conducive to the rearrangement of polymer chains and the formation of regular and orderly crystalline structures [13]. Less crystallinity meant that less polymer molecular chains were compact and it was less difficult for drug molecules to cross over the polymer molecular chains.

The drug release rates of P34HB-10 coatings were faster than P34HB-1 under the same polymer concentrations. The main reason for this may be that P34HB is a thermoplastic crystalline polymer material, and its crystallinity varies with the content of 4HB. A higher content of 4HB will lead to a lower crystallinity [14]. Therefore, the drugs in P34HB-10 could pass through the polymer coatings more easily, due to its lower crystallinity than P34HB-1.

When the concentration of spraying solution is too low, the spraying process will take a long time, which will greatly affect the processing efficiency. An 18 mg/mL solution takes only half the spraying time compared to an 8 mg/mL solution to form a stent coating, thus greatly improving the processing efficiency. Considering the spraying efficiency and drug release effect, an 18 mg/mL solution of P34HB was selected for the subsequent experiments.

### 3.2. Effects of Drug Polymer Ratios Prepared for Coatings on Drug Release Rates

The experiment in this section was used to investigate the effects of spraying solutions with different drug polymer ratios, which were prepared for coatings, on drug release rates, while these solutions retained the same polymer concentrations (such as 18 mg/mL).

**Figure 3:**
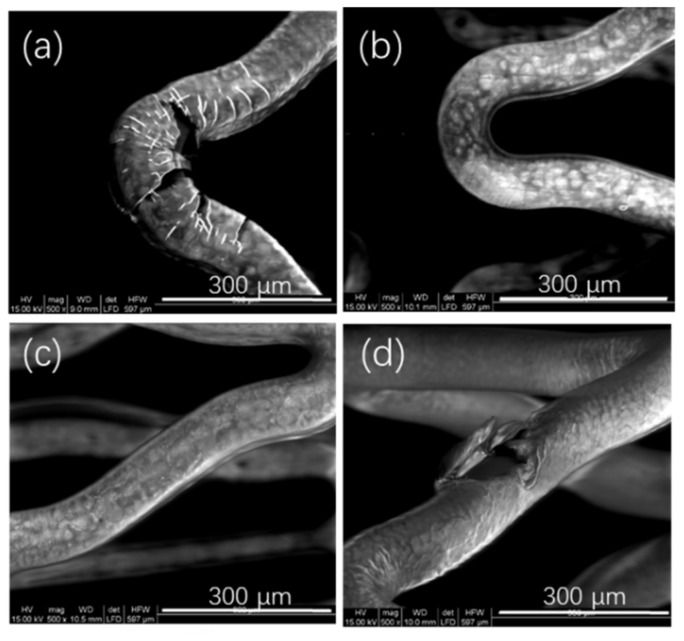
Morphologies of coating surfaces made with different drug polymer ratios of P34HB. (**a**) 1:1; (**b**) 1:2; (**c**) 1:10; (**d**) 1:12.

When the drug polymer ratio was 1:1 (Figure 3a) or even more, the locations with larger deformation of the stents after stents were expanded began to have some puckers or fissures. With the gradual decrease of drug polymer ratio, the coatings became smoother and smoother. Owing to the flexibility of the polymer, higher concentrations of the polymer in the polymer matrix allowed better flexibilities for drug coatings, i.e., a better deformation performance of coatings could be obtained [12]. When the drug polymer ratio was 1:12 (Figure 3d) or even lower, the weights of the coatings would be relatively large, causing the coatings to crack after the expansion of the stents. Therefore, the investigated range of drug polymer ratios in this experiment was from 1:2 (Figure 3b) to 1:10 (Figure 3c).

**Figure 4:**
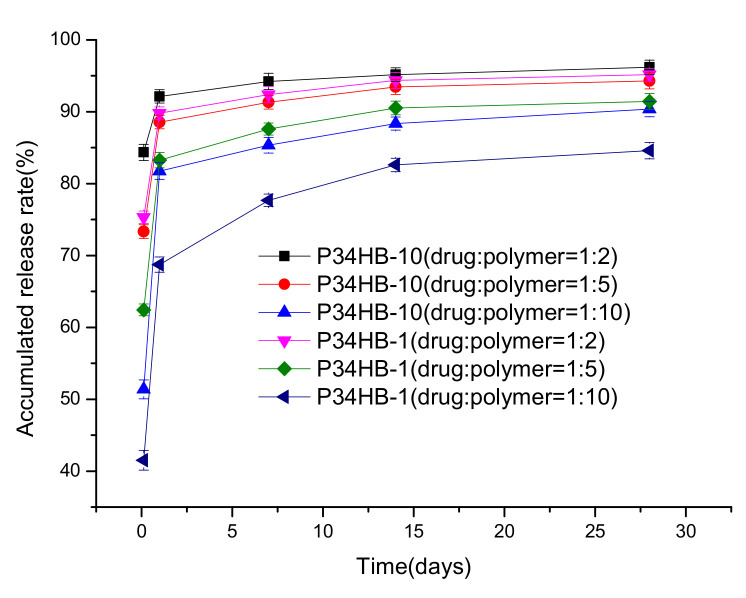
Drug release profiles of coatings with different drug polymer ratios.

Figure 4 shows that both the P34HB-1 and P34HB-10 coatings’ drug release rates increased as the polymer solutions’ drug polymer ratios changed from 1:10 to 1:2. The release profiles of the drugs in the polymer coatings were controlled by the drug diffusion [12,13,14,15]. The polymer coatings became swollen and the drugs could diffuse from the coatings into the medium solutions. The coatings with high drug concentrations presented high release rates, owing to the high drug diffusion rates [12].

The drug release rates of P34HB-10 coatings became faster than P34HB-1 under the same drug polymer ratios. As seen in Figure 4 above, this may have been because the 4-HB number in P34HB-10 is higher than in P34HB-1, so drugs in P34HB-10 could more easily pass through the polymer coatings.

P34HB-1 coatings prepared with a 1:10 drug polymer ratio showed good drug sustained release effects, while the other ratios did not; thus, P34HB-1 may be a latent polymer for DES coatings.

### 3.3. Effects of Spraying Parameters on Coating Drug Release Rates

#### 3.3.1. Factor Levels and Response Variable Design

There were three main spraying parameters, including Rate of solution flow, Mandrel movement speed, and Focusing pressure, that affected the P34HB-1 coatings’ drug release rate in the preliminary experiments.

**Figure 5:**
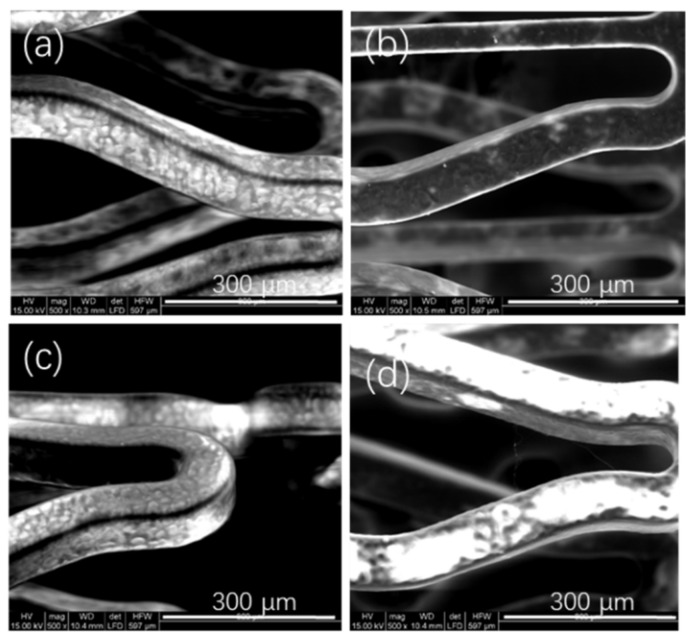
Morphologies of coating surfaces made with different Rates of solution flow of P34HB-1. (**a**) 0.01 mL/min; (**b**) 0.02 mL/min; (**c**) 0.08 mL/min; (**d**) 0.09 mL/min.

When Rate of solution flow was 0.01 mL/min (Figure 5a) or less, the P34HB-1 coatings began to become unsmooth during spraying. When Rate of solution flow reached 0.09 mL/min (Figure 5d) or more, filaments appeared in the places with a larger deformation of the stents during spraying. Thus the coatings with Rates of solution flow between 0.02 mL/min (Figure 5b) and 0.08 mL/min (Figure 5c) are smooth and have no filaments.

When Mandrel movement speed was 0.1 cm/s or less, filaments began to appear at the places with a larger deformation of the stents during spraying. When Mandrel movement speed reached 0.9 cm/s or more, the P34HB-1 coatings began to become unsmooth during spraying. The filaments and roughness here were the same as above. Thus the coatings with Mandrel movement speeds between 0.2 cm/s and 0.8 cm/s are smooth and have no filaments.

When Focusing pressure was 0.4 cm/s or less, the coatings sprayed on the stents began to become rough, due to the small quantities during spraying. The roughness here was the same as above. When Focusing pressure reached 2.6 cm/s or more, the distal ends of the stents shook during the spraying process, resulting in different coating weights at various parts of the stents. Thus the coatings with Focusing pressures between 0.5 cm/s and 2.5 cm/s are smooth and even.

Since the drug release of a 24-h release point is relatively stable and is the key release point affecting the whole release profile, the 24-h release point was selected as the key response variable to investigate the effects of spraying parameters on coating drug release rates.

Table A1 (Appendix A) shows the limit level values required for the DOE of the three influencing factors for the response variable (24 h drug release rate).

#### 3.3.2. DOE Design and Analysis

P34HB-1 coatings were prepared with 18 mg/mL polymer concentration solutions with a 1:10 drug polymer ratio. Table A2 (Appendix A) shows the DOE of the effects of the spraying parameters on the coating drug release rates.

**Figure 6:**
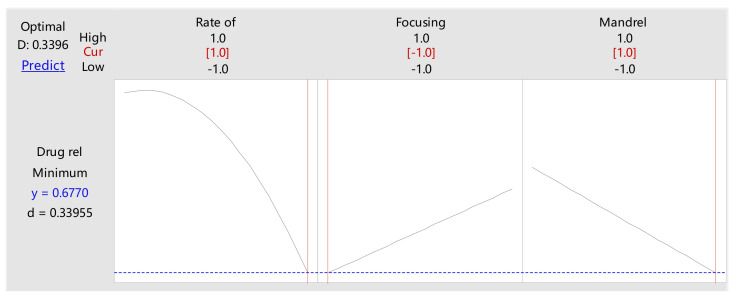
The response optimizer of DOE.

The DOE adopted the response surface regression method for data analysis, and the analysis results are shown in Table 1 and Table 2 and Figure 6 below:

Table 1 shows that the *p* value of the fitted model was equal to 0.000, less than 0.05; the *p* value of the lack-of-fit for the model was equal to 0.114, more than 0.05. Table 2 shows that R-sq (98.32%) was very close to R-sq (adj) (96.64%), and both were very close to 100%. Thus, the results of both Table 1 and Table 2 demonstrate that the model fit was valid.

The response optimizer in Figure 6 gives the change trends and optimal values of all three influence factors and corresponding response variable: the 24 h drug release rates of P34HB-1 coatings decreased as Rates of solution flow increased; the 24 h drug release rates of P34HB-1 coatings decreased as Focusing pressures decreased; the 24 h drug release rates of P34HB-1 coatings decreased as Mandrel moving speeds increased. When both Rate of solution flow and Mandrel moving speed increased to a high level (0.08 mL/min, 0.8 cm/s), Focusing pressure decreased to a low level (0.5 cm/s) and the 24 h drug release rates reached a minimum value of 0.6770 (67.7%).

Since the 24 h drug release rate is the key release point affecting the whole release profile, the influence trends of the three influencing factors on the whole drug release profile are the same as for the 24 h drug release rate.

**Figure 7:**
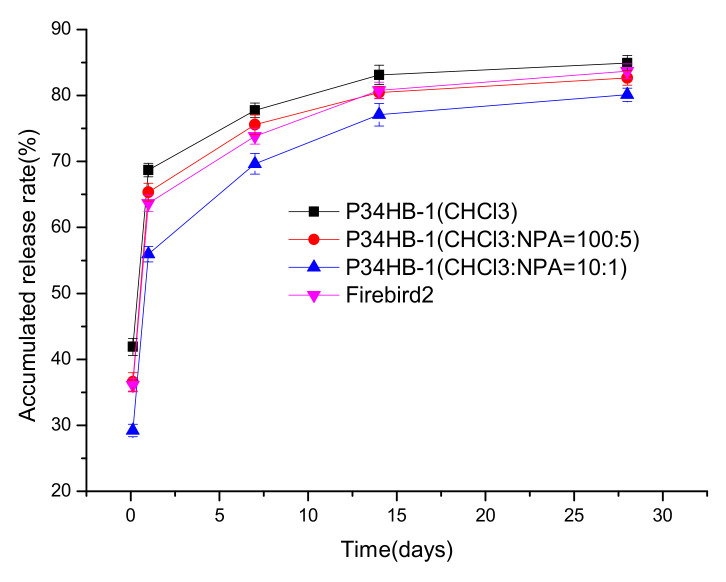
Drug release profiles of P34HB-1 DES and Firebird2^®^.

Three stents were individually sprayed with P34HB-1/CHCl_3_ solution under the optimal values of the three spraying parameters, to obtain the drug release curves of the coatings, as shown in Figure 7, which were faster than Firebird2^®^, and the 24 h drug release rate was 68.7%; relatively close to 67.7%. Thus, the controlled release rates for P34HB-1 coatings became more important.

### 3.4. Mixed Solvents Effects on Drug Release Rates

When the molecular weight of a polymer remains unchanged, improving the close arrangement of the molecular structure may help to reduce the diffusion rate of drugs in the polymer. Therefore, this is a very important direction to explore, to find a suitable process method to improve the crystallinity of a polymer.

The relevant literature [16] shows that two solvents with similar polarities induce PLA to produce a higher crystallinity more easily than a single solvent. Mixed solvents with a similar polarity can effectively promote the movement of polymer molecular segments, make these segments regularly arranged and aggregated, promote the growth of crystalline chains, expand the area of the crystalline region, and improve the crystallinity. The greater the polarity difference of the mixed solvents, the lower the crystallinity. Here, NPA with a similar polarity was added to the best solvent CHCl_3_ of P34HB-1, to form P34HB-1/drug solutions for the experiments. The polarities of CHCl_3_ and NPA were 4.4 and 4.0, respectively. When the ratio of CHCl_3_:NPA reached 10:2 or more, the P34HB-1 solutions began to turn turbid. Therefore, the investigated range of ratios of CHCl_3_:NPA in this experiment were within 10:1.

**Figure 8:**
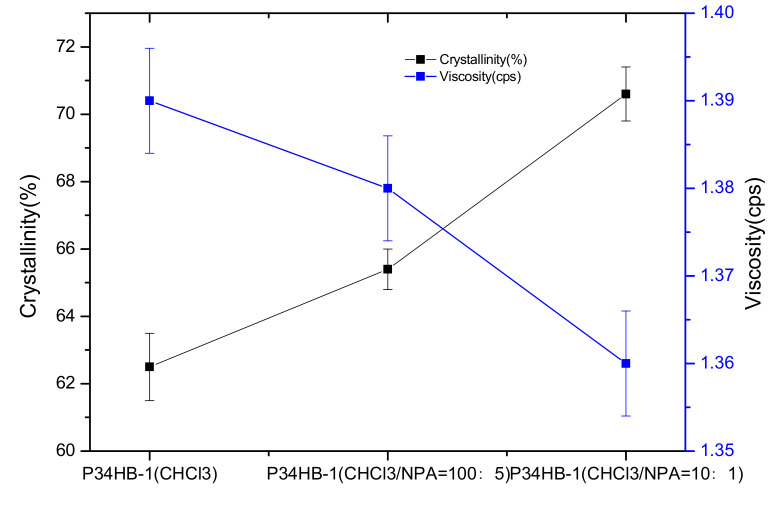
Crystallinity and viscosity of P34HB-1.

As shown in Figure 7, with the gradual increase of NPA contents in the mixed solvents, the drug release rates of the P34HB-1 coatings decreased gradually, within 28 days. When CHCl_3_:NPA = 10:1, the drug release rates were significantly lower than Firebird2^®^. The main reason for this may have been the higher crystallinity of P34HB-1 in the mixed solvent (CHCl_3_:NPA = 10:1) than in the mixed solvent (CHCl_3_:NPA = 100:5) and pure CHCl_3_. Figure 8 shows that with the gradual increase of NPA contents in the mixed solvents, the crystallinity of the polymer increased gradually. This suggests that there was a more orderly accumulation of polymer molecular segments in P34HB-1 with the mixed solvents, and thus the coatings became more compact. P34HB-1 had a lower viscosity in mixed solvents than in pure CHCl_3_, as shown in Figure 8, and this lower viscosity may have helped to increase the crystallinity.

P34HB-1 coatings had better controlled drug release rates in mixed solvents (CHCl_3_:NPA = 10:1) than Firebird2^®^, thus P34HB-1 is a latent polymer for DES.

### 3.5. Effects of Double-Layer Coatings on Drug Release Rates

In addition to the mixed solvents effects method, the multi-layer coating structure method may effectively improve the drug release rates of coatings, especially when a controlled-release outer layer without drugs is introduced. The drug coatings of stents can have two or three layers, according to requirements; for example, the Cypher^®^ coating has three layers: Parylene C as a supporting layer ensures good adhesion between the coating and stent, and the outermost layer (PEVA/PBMA) does not contain drugs, ensuring a good drug release rate [15]. As the P34HB-1, here, had a good adhesion throughout the preliminary experiments, a two-layer coating was designed: the inner layer was prepared using a P34HB-1/RAPA solution with CHCl_3_, and the outer layer (C) was P34HB-1.

When the weights of C in this experiment reached 860 μg or more, the coatings cracked easily after the stents were expanded, because they were too thick. This cracking of the coatings is shown in Figure 3. Therefore, the maximum weight of outer layer investigated in this experiment was 800 μg.

**Figure 9:**
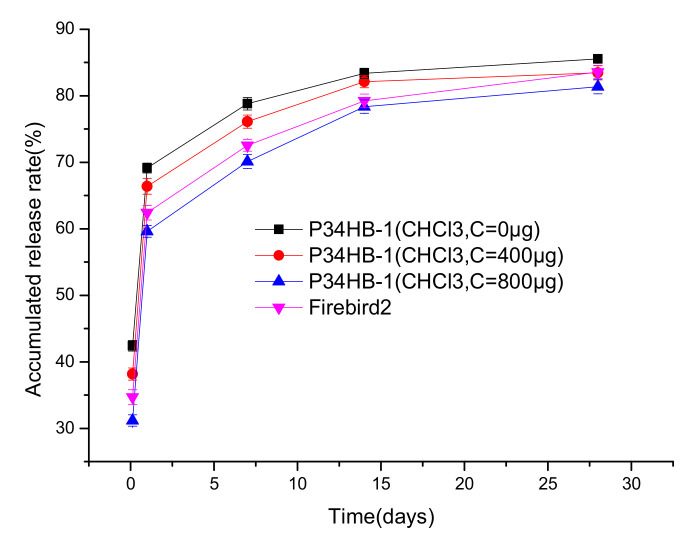
Drug release profiles of P34HB-1 (different outer layer contents) and Firebird2^®^.

Figure 9 shows that the drug release rates of the P34HB-1 coatings gradually decreased when the C weights were increased from 0 to 800 μg, and when the inner layers retained the same contents. This showed that the controlled-release layers were helpful to control the drug release rates. With the increase of the thickness of C, the time required for polymer swelling and the paths required for drug release become longer, so the rate of drug release slowed down [17,18]. When the C weight reached 800 μg, the drug release rate was slower than Firebird2^®^. Thus, the effect of increasing the weights of the controlled-release layers, to reduce the drug release rate, was promising.

### 3.6. Effects of Both Mixed Solvents and a Double-Layer Structure on Drug Release Rates

**Figure 10:**
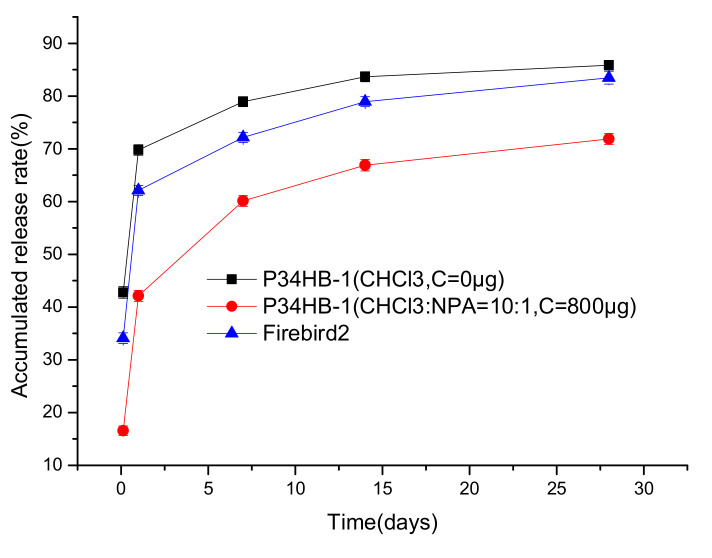
Drug release profiles of P34HB-1 (both mixed solvents and double layers) and Firebird2^®^.

Since both the mixed solvents method and double-layer structure method are effective for controlling coating drug release rates, it was very important to consider the effects of mixed solvents and a double-layer structure, together, on the drug release rate. A two-layer coating was designed here: the inner layer was prepared with a P34HB-1/RAPA solution with CHCl_3_/NPA solvents, and the outer layer (C) was P34HB-1. Figure 10 shows that the P34HB-1 coatings prepared with both mixed solvents and double layers had far more effectively controlled drug release rates than Firebird2^®^. These effects were also greater than the P34HB-1 coatings prepared with only mixed solvents or double layers, as shown in Figure 7 and Figure 9.

The model fitted in this experiment for the P34HB-1 coatings prepared with both mixed solvents and double layers, as in Equation (2), had the best fit value (adj.R^2^ = 0.994); therefore, this was an effective model to combine with a diffusion-relaxation model and a corrosion (Ritger–Peppas) model, to study the drug release kinetics [15]. The coefficient of x^1/2^ is related to pure Fick diffusion, the coefficient x is related to the phenomena of corrosion and relaxation, and the coefficients x^2^ and x^3^ are related to the phenomenon of corrosion. The coefficient values of both x^1/2^ (56.93278) and x (−16.2357) were so much larger than the others, that the diffusion factor and relaxation (swelling) factor were dominant, which explains the first sudden increase and then the slow increase of P34HB-1′s drug release profile in Figure 10. The coefficient values of both x^2^ and x^3^ were so small that they were negligible, thus the corrosion factor might have had little contribution to the drug release of DES.

y = 56.93278x^1/2^ − 16.2357x + 0.54003x^2^ − 0.00903x^3^ (2)

P34HB-1 coatings prepared with both mixed solvents and double layers had more effectively controlled drug release rates compared to P34HB-1 coatings prepared with only mixed solvents or double layers and had far more controlled drug release rates than Firebird2^@^ within the whole release period, from 0 to 28 days; suggesting that P34HB-1 stents prepared with both mixed solvents and double layers had a more effective control for a longer drug release period. Therefore, P34HB-1 is a latent, suitable polymer for drug release coatings.

## 4. Conclusions

Both P34HB-1 and P34HB-10 coatings showed increased drug release rates as the polymer concentrations were gradually increased from 8 mg/mL to 28 mg/mL. Both P34HB-1 and P34HB-10 coatings showed increased drug release rates as the drug polymer ratios were gradually changed from 1:10 to 1:2. The drug release rates of the P34HB-1 coatings became slower than P34HB-10, thus showing sustained drug release effects. The drug release rates of the P34HB-1 coatings decreased as Rates of solution flow increased, decreased as Focusing pressures decreased, and decreased as Mandrel moving speeds increased. The P34HB-1 coatings prepared with CHCl_3_/NPA (10:1) mixed solvents had better controlled drug release rates compared to Firebird2^®^. The drug release rates of P34HB-1 coatings prepared with CHCl_3_ solutions decreased as the outer layer weights were increased from 0 to 800 μg. When the outer layer weight reached 800 μg, the drug release rate of the P34HB-1 coating was slower than Firebird2^®^. P34HB-1 coatings prepared with both CHCl_3_/NPA (10:1) mixed solvents and double layers had more effectively controlled drug release rates than P34HB-1 coatings prepared with only mixed solvents or double layers and these effects were far greater than Firebird2^@^, thus P34HB-1 is a latent polymer for DES.

## Figures and Tables

**Table 1:** Analysis of Variance.

Source	DF	Adj SS	Adj MS	F-Value	*p*-Value
Model	5	0.006541	0.001308	58.58	0.000
Linear	3	0.005930	0.001977	88.51	0.000
Rate of solution flow	1	0.003784	0.003784	169.46	0.000
Focusing pressure	1	0.001568	0.001568	70.21	0.000
Mandrel moving speed	1	0.000578	0.000578	25.88	0.004
Square	1	0.000190	0.000190	8.51	0.033
Rate of solution flow*Rate of solution flow	1	0.000190	0.000190	8.51	0.033
2-Way Interaction	1	0.000421	0.000421	18.83	0.007
Rate of solution flow*Focusing pressure	1	0.000421	0.000421	18.83	0.007
Error	5	0.000112	0.000022		
Lack-of-fit	3	0.000103	0.000034	7.92	0.114
Pure Error	2	0.000009	0.000004		
Total	10	0.006653			

**Table 2:** Model Summary.

S	R-sq	R-sq (adj)	R-sq (pred)
0.0047258	98.32%	96.64%	88.70%

## Data Availability

Data are available on request, due to restrictions e.g., privacy or ethical considerations. The data presented in this study are available on request from the corresponding author. The data are not publicly available due to later possible commercial uses.

## Appendix A

**Table A1:** Table of factor level values.

Factors Names/Factors Level Values	−1	+1
Rate of solution flow	0.02 mL/min	0.08 mL/min
Mandrel moving speed	0.2 cm/s	0.8 cm/s
Focusing pressure	0.5 cm/s	2.5 cm/s

**Table A2:** DOE of the effects of spraying parameters on the coating drug release rates.

StdOrder	RunOrder	CenterPt	Blocks	Rate of Solution Flow	Focusing Pressure	Mandrel Moving Speed	Drug Release Rate (24 h)
7	1	1	1	−1	1	1	74.6%
1	2	1	1	−1	−1	−1	72.6%
8	3	1	1	1	1	1	69.0%
9	4	0	1	0	0	0	72.4%
11	5	0	1	0	0	0	72.1%
4	6	1	1	1	1	−1	70.8%
10	7	0	1	0	0	0	72.5%
6	8	1	1	1	−1	1	68.3%
3	9	1	1	−1	1	−1	76.8%
2	10	1	1	1	−1	−1	68.8%
5	11	1	1	−1	−1	1	70.3%
